# The Cure of Bright's Disease by Operation

**Published:** 1902-01-11

**Authors:** 


### THE CURE OF BRIGHT'S DISEASE BY OPERATION.

Dr. George Edebohls, of New York, proposes to cure chronic Bright's disease by stripping off and removing the capsule of the kidney—in fact, he says he has done it, and he speaks of the operation as one which is to be recommended in such cases. His first suggestion in this direction was made in 1899 and was the result of his observing how great an improvement in the general condition had followed the operation in four out of six cases in which he had performed nephropexy for the purpose of anchoring a movable kidney in the presence of well-marked chronic Bright's disease. In 1898, however, he did an operation "with the deliberate purpose of curing chronic Bright's disease," and "as a result of the operation the patient was radically cured . . . and remains so to this day." For a while the suggestion that the cure of the Bright's disease which occurred in the cases operated on might be due to the correction of the diplacement of the kidney by the nephropexy, stood in the way of further development of the idea; but when three secondary operations, upon kidneys which had been anchored some time previously, demonstrated the cure of chronic Bright's disease by operation, he began to see his way more clearly.

Dr. Edebohls[1] now gives an account of 18 cases in which he has operated for chronic Bright's disease. All were women. In 14 of them both kidneys were operated on, in 12 instances at one sitting and twice at two sittings, while in four patients the operation was performed on one kidney only, this being in every instance on the right side, and he states that of the four patients in whom the right kidney alone was operated on two have completely recovered. "It may be taken for granted then that their left kidneys were at the time of operation, as they are now, in perfect health." This brings us to another point which Dr. Edebohls has discovered, to his own surprise as it certainly is to ours, namely, that chronic Bright's disease is often unilateral. "In six of the eight cases in which chronic Bright's disease is recorded as unilateral, the healthy condition of the other kidney was verified at operation when both kidneys were brought out of the wound for careful and critical examination." This unilaterality of the disease seems to Edebohls to explain the chronic course of many cases of chronic Bright's disease, and the comparatively little disturbance of the health sometimes occasioned. The healthy kidney does the work and toxic symptoms do not arise. Gradually, however, "the steady loss of albumin through the affected kidney tells upon and undermines the general health." Probably also, towards the end, the other kidney generally becomes involved, "thus explaining the very great preponderance of bilateral over unilateral chronic Bright's disease as found in the deadhouse."

There is no doubt that surgeons have taught pathologists a good deal during recent years, but this that we are now told as to the unilaterality of chronic Bright's disease, and of the curability, even by operation upon one kidney alone, of a disease of such a widespread character as chronic interstitial nephritis has come to be regarded, goes a long step beyond anything that surgery has hitherto attempted. Probably the pathologists will make short work of Dr. Edebohls. If not we shall all be taken back even beyond the days of our youth, back to the days when Gull and Sutton made war upon Dr. Johnson, to the days when arterio-capillary fibrosis was a new thing, and Bright's disease was regarded as a disease of the kidney alone, all the rest being secondary to it. This would be a serious upsetting of all modern teaching, but if we can cure the malady by operation there would seem to be no other way out of the difficulty, unless, indeed, a little healthy scepticism as to the diagnosis might afford a means of escape from the dilemma. Even this resource, however, seems to be closed to us, for we are told that "the diagnosis of chronic Bright's disease in the above 18 cases was based upon the previous history of the patient, upon the chemical and microscopical examination of the urine, and, lastly, upon the critical test of actual inspection and palpation of the kidney at the time of operation. This evidence was supplemented in two cases by microscopical examination of a small piece of kidney tissue removed at the operation." The delivery of the kidney upon the skin of the loin enabled Dr. Edebohls, we are told, to demonstrate to all witnesses of each operation the visible changes produced by chronic Bright's disease in the particular kidney under operation—the adherent capsule, nodulation, granular condition of the subcapsular surface, shrinking, unequal contraction, and occasional cyst formation of chronic interstitial nephritis, and the various changes due to chronic parenchymatous nephritis, including "cloudy swelling." So there is nothing left for us to do but to reform our pathology. It must be added that if our pathologists wish to make themselves really useful, they must be prepared to arrive at a conclusion as to the conditions met with while the operation is going on, as they do in America. For example, in one case we are told that, as the kidney lay delivered and stripped of its capsule proper, Dr. Edebohls suspected the presence of a diffuse tuberculosis, so a minute piece of kidney substance was exsected and taken to the laboratory for examination, the kidney meanwhile being replaced within the body while the operation was being continued on the other side. "In 15 minutes the report came from Dr. Brooks: 'Chronic interstitial nephritis; no tuberculosis': whereupon nephropexy upon both kidneys was completed." In the operation performed by Dr. Edebohls the kidney is first delivered through the wound and freed from its fatty capsule, and then the capsule proper is divided on a director along the entire length of the convex external border of the kidney, "and clean around the extremity of either pole." Each half of the capsule proper is in turn stripped from the kidney and reflected towards the pelvis until the entire surface of the kidney lies raw and denuded before the operator. The stripped-off capsule is then cut away entirely, close to its junction with the pelvis of the kidney, and removed. The kidney is then dropped back into its fatty bed and the external incision is closed. After both kidneys have been thus operated upon the dressings are applied and the patient is put to bed. It is quite evident that Dame Nature in constructing the human frame would have been well advised to have given us our organs without their capsules.

[1] New York Med. Rec., Dec. 21.

